# Cataract among Patients with Renal Transplantation in a Tertiary Care Centre: A Descriptive Cross-sectional Study

**DOI:** 10.31729/jnma.7946

**Published:** 2023-01-31

**Authors:** Madhu Thapa, Gulshan Bahadur Shrestha, Pragati Gautam, Mahesh Raj Sigdel

**Affiliations:** 1 Department of Ophthalmology, Maharajgunj Medical Campus, Tribhuvan University Teaching University, Maharajgunj, Kathmandu, Nepal; 2 Department of Nephrology, Maharajgunj Medical Campus, Tribhuvan University Teaching University, Maharajgunj, Kathmandu, Nepal

**Keywords:** *cataract*, *prevalence*, *renal transplantation*, *steroid*

## Abstract

**Introduction:**

Intensive immunosuppressant therapy after renal transplantation has found to cause many ocular sides effects, cataract being one of the common one.. Studies on a similar topic have still remained explored in our setting. The aim of the study was to find out the prevalence of cataract among patients with renal transplantation in a tertiary care centre.

**Methods:**

This descriptive cross-sectional study was conducted among patients of renal transplantation at tertiary care centre from 1 May 2021 to 31 October 2021. The data was collected after the ethical approval from Institutional Review Committee [Reference number: 397(6-11) e2077/078]. Study proforma recorded the number of patients with cataracts, duration of steroid use, mean age and other comorbidities. A convenience sampling method was used. Point estimate and 95% Confidence Interval were calculated.

**Results:**

Out of 31 renal transplant patients, 10 (32.26%) (15.80-48.72, 95% Confidence Interval) had cataract.

**Conclusions:**

The prevalence of cataract among renal transplantation patients was found to be lower than similar studies done in similar settings.

## INTRODUCTION

Renal diseases are on the rise globally due to an increase in cases of non-communicable diseases like diabetes and hypertension. Besides them, chronic glomerulonephritis, obstructive urinary tract diseases, polycystic kidney diseases, connective tissue diseases, use of certain drugs like nonsteroidal anti-inflammatory drugs, and antiretroviral-agents, can also lead to compromised renal function.^1^ Renal transplant is the only definite treatment for endstage renal disease (ESRD).^2^ There is a significantly high chance of rejection following renal transplant, so long-term intensive immunosuppressive therapy is given following the surgery. Triple therapy for immunosuppression includes Mycophenolate mofetil (or azathioprine) and tacrolimus (or Cyclosporine) and corticosteroid.^3^

Corticosteroid causes significant ocular side effects, mainly cataract formation and glaucoma which leads to decreased vision in these patients when used for a long duration. Timely identification of ocular morbidity and proper management of those conditions can save the eyesight of these patients.

The study aimed to find out the prevalence of cataract among patients with renal transplantation in a tertiary care centre.

## METHODS

This descriptive cross-sectional study was done among patients who had undergone renal transplantation in Tribhuvan University Teaching Hospital (TUTH) from 1 May 2021 to 31 October 2021. Ethical approval was taken from the Institutional Review Committee of the Institute of Medicine [Reference number: 397(6-11) e2077/078]. Patients who had renal transplantation at least three months prior were referred from Nephrology Department, TUTH, for ophthalmic evaluation at B. P. Koirala Lions Centre for Ophthalmic Studies (BPKLCOS), Department of Ophthalmology, Institute of Medicine were included in the study. Patients with a history of significant ocular morbidities before renal transplant were excluded. Informed consent was taken from all the subjects. After informed consent was taken, all patients were examined in detail in the out-patients department of BPKLCOS. A convenience sampling method was used. The sample size was calculated using the formula:


$$\begin{array}{ll} \text{n} & ={\text{Z}}^{2}\times \frac{\text{p}\times \text{q}}{{\text{e}}^{\text{2}}}\\ & =1.96^{2}\times \frac{0.50\times 0.50}{0.10^{2}}\\ & =96 \end{array}$$

Where,

n = minimum required sample size
Z = 1.96 at 95% Confidence Interval (CI)
p = prevalence taken as 50% for maximum sample size calculation
q = 1-p
e = margin of error, 10%

The sample was adjusted for the finite population as follows:


$$\begin{array}{ll} \text{n} & =\text{n }[1+\{\frac{\text{(n}-1)}{\text{N}}\}]\\ & =\text{n }[1+\{\frac{\text{(96}-1)}{\text{31}}\}]\\ & =24 \end{array}$$

Where,

n'= adjusted sample size

N = 31, finite population

The calculated minimum required sample size was 24. However, a total of 31 sample size was taken.

A cataract is the degradation of the optical quality of the crystalline lens that affects the vision.^4^ Any lenticular opacity was classified as a cataract. Along with this, duration of steroid use, renal function and coexisting co-morbidities were also recorded.

Data were entered and analysed in IBM SPSS Statistics version 21.0. Point estimate and 95% CI was calculated.

## RESULTS

**Figure 1:**
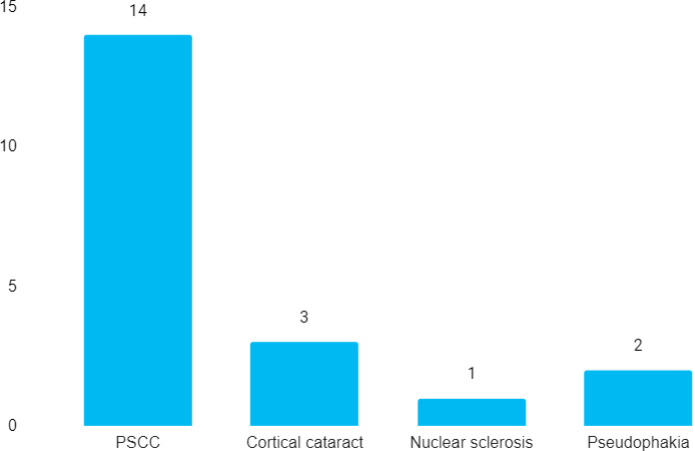
Types of cataract (n= 20).

Out of 31 renal transplant patients, 10 (32.26%) (15.8048.72, 95% CI) had cataracts in both eyes. The post-subcapsular cataract (PSCC) was the commonest type of cataract seen in 14 (70%) eyes (Figure 1).

**Figure 2:**
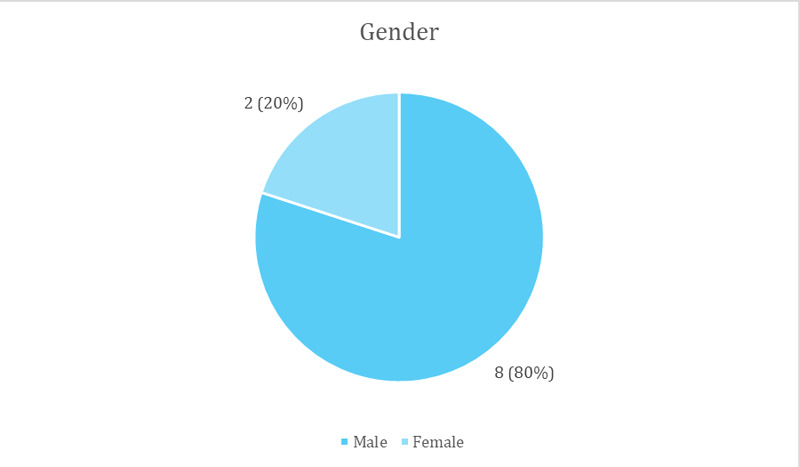
Genderwise distribution of cataracts among renal transplant patients (n= 10).

Their mean age was 43±12.78 years ranging from 19 years to 62 years. The mean duration of renal transplant among patients with cataracts was 7±3.88 years ranging from three months to 13 years. The subnormal visual acuity (equal or less than 6/9) was seen in 12 (60%) in the right eye and 8 (30%) in the left eye among cataract cases. Out of them, 2 (10%) patients had already undergone cataract surgery in one eye each (Figure 2).

Out of 10 patients who developed cataracts, the mean duration of use of the oral corticosteroid was 9.10±4.93 years ranging from 3 years to 17 years.

In patients with cataracts, 7 (70%) had coexisting hypertension whereas only 2 (20%) had diabetes and 1 (10%) of the patients had a recurrent history of urinary tract infection. Out of them only 2 (20%) of patients were suspicious of glaucomatous optic disc changes but intraocular pressure (IOP) and automated visual field tests were within normal limit. Out of them, 6 (60%) of patients had an abnormal renal function and 4 (40%) had a normal renal function.

All 10 (100%) patients were on oral steroids following renal transplant. A total of 8 (80%) were on 5 mg and less daily dose of steroid whereas 3 (30%) were on more than 5 mg daily dose. Renal function status was determined by serum creatinine value with the cut-off value of 62-115 micromoles per litre (Table 1).

**Table 1:** Renal function status (n= 10).

Renal function status	n (%)
Abnormal	6 (60%)
Normal	4 (40%)

## DISCUSSION

In this study, the prevalence of cataracts among the patients who underwent renal transplantation was 32.26%. Long-term steroid has been associated with a strong correlation with cataracts. In our study 32.26% of subjects had cataracts. Among them, PSCC was seen in about 70%. A similar incidence of PSCC has been found in other studies as well.^5-7^ A report from Ethiopia has reported a much less incidence of cataracts in their population.^8^ On contrary, an Indian study has reported a much higher incidence (40%) of cataracts among patients with renal failure.^9^ The mean use of steroids was 9.10±4.932 years ranging from 3 to 17 years, suggesting that the longer the duration of use of steroids more is the chances of cataract formation.

The mean age was 43±12.78 similar to a published report.^5^ Some studies have reported the mean age of patients to be 53.4 years,^10^ 59±11 years,^11^ which is higher than our finding. Less than 40 years are found to have fewer chances of progressing to ESRD.^12^ Among patients who had developed cataracts, 80% were males. This might also suggest that in our maledominated society, males go tend to go for live-saving procedures more than females. The mean duration since renal transplant was 7±3.88 years ranging from 3 months to 13 years. Hypertension was found as a significant coexisting condition risk factor, accounting for 70% of the cases and Diabetes was seen in 20% of the cases only. Among the patients who had developed cataracts, 60% of patients had an abnormal renal function and 40% had a normal renal function. Serum creatinine level predicts long-term graft survival.^13^ Increased serum creatinine level might indicate an increase in ocular complications due to increased chances of hypertensive retinal changes as well as cataracts due to electrolyte dis-balance.

Similar to other published literature, sub-normal visual acuity of less than 6/9 was seen in 60% of the right eye and 50% in the left eye among the cases of cataracts.^5,11^ Better visual acuity suggests fewer ocular abnormalities. Renal transplant improves renal function and systemic as well as ocular complications. That may be one of the reasons that our patients had good visual acuity. The mean age of our patients is relatively young which may also contribute to less visually threatening retinal complications which are frequently seen more in older age groups.^14^

Steroid-induced glaucoma is another important side effect of chronic steroid use in these patients.^15^ Among the cases that had cataracts, 20% of cases had suspicious glaucomatous optic neuropathy, but IOP and automated visual field test were was normal in all cases. This may suggest that as the incidence of steroid-related cataracts increases the chances of steroid-induced glaucoma also increases. Published article has reported 5% glaucoma among all renal transplant patients,^10^ whereas others did not find any cases of glaucoma in their study.^5^ Only 1.4% of patients had increased IOP in a published study.^7^ Cases of increased IOP is less common when the patient is on oral steroid as compared to the patients on topical steroid. That may be the reason for less glaucoma in our study population.^16^

The limitation of the study includes the small sample size and sampling method. Due to the descriptive nature of the study, further association been ocular findings and steroid use, a dose of steroid use could not be established.

## CONCLUSIONS

The prevalence of cataracts among renal transplantation patients was found to be lower than in published literature. Various ocular morbidities may present among renal transplant patients on oral steroids. Thus, the routine ocular examination is recommended among renal transplantation patients who are on long-term steroids.
